# Leniency Bias in Performance Ratings: The Big-Five Correlates

**DOI:** 10.3389/fpsyg.2017.00521

**Published:** 2017-04-10

**Authors:** Kevin H. C. Cheng, C. Harry Hui, Wayne F. Cascio

**Affiliations:** ^1^Innushuk ConsultSydney, NSW, Australia; ^2^Department of Psychology, University of Hong KongHong Kong, Hong Kong; ^3^CU Denver Business School, University of Colorado at DenverDenver, CO, USA

**Keywords:** rating leniency, work performance, personality, extroversion, Agreeableness, Big Five personality traits, self-rating, others rating

## Abstract

Some researchers assume that employees' personality characteristics affect leniency in rating others and themselves. However, little research has investigated these two tendencies at the same time. In the present study we developed one index for other-rating leniency and another one for self-rating leniency. Based on a review of the literature, we hypothesized that a generous assessment of peers would more likely be made by those who are extroverted and agreeable than by those who are not. Furthermore, a generous assessment of oneself would more likely be made by people who are conscientious and emotionally stable, than by people who are not. We also investigated if the leniency in rating others and the leniency in rating oneself are part of a more general leniency tendency. Data collected from a sample of real estate dealers provided support for the above hypotheses. Limitations and implications for future research are discussed.

## Introduction

### Dispositional factors undermining accuracy of work performance

Theoretically, a performance rating embodies the following: the ratee's level of work performance, the rater's perspective (i.e., self, peer, supervisor, etc.), the rater's idiosyncrasies, and random error. Using managers' developmental rating data, Scullen et al. (2000) found that the ratee's actual performance accounted for only about 20–25% of the variability in performance ratings when averaged across dimensions, perspectives, and instruments. Similar studies agreed that the influence on performance ratings for all combinations of rater perspectives and performance dimensions originated from rater's bias (Castilla and Benard, 2010; Ng et al., 2011; Bono et al., 2012). One important component of the bias is a rater's leniency.

On the basis of reviews on the link between personality and organizational behavior (Barrick and Mount, 1991, 2005; Barrick et al., 2001; Ones et al., 2007; Shaffer and Postlethwaite, 2013), we adopt the Big Five model for a better understanding of the personality substratum of rating leniency (Ceschi et al., 2016). The primary premise of this study is that certain personality dimensions of the Big Five are related to leniency in the ratings one assigns to oneself and others. In the following sections we elaborate on what might define this relationship.

### Possible causes of rating bias when rating others, and the role of personality

Why would some people rate their subordinates leniently, that is, assign performance ratings that are more generous than might be justified? More than half a century ago, Thorndike (1949) and Glickman (1955) identified eight possible explanations. In particular, the rater: (1) may feel that anyone under his or her jurisdiction who is rated unfavorably will reflect poorly on the rater's own worthiness (Paulhus, 1984); (2) may feel that anyone who could have been rated unfavorably had already been discharged from the organization; (3) may feel that a derogatory rating may be revealed to the ratee to the detriment of relations between rater and ratee; (4) may rate leniently in order to win promotions for his or her subordinates and therefore indirectly increase the rater's future control of those subordinates by earning a reputation as a supervisor with “influence upstairs;” (5) may be projecting; (6) may feel it necessary always to approve others in order to gain approval for himself or herself (Wayne and Liden, 1995); (7) may be operating on the basis: “Whoever associates with me is meritorious, therefore I am meritorious;” and (8) may rate leniently because there exists in the culture a response set to approve rather than disapprove. Other reasons can be found in Fleenor et al. (2010) and Reich et al. (2007).

Some of these possible reasons for leniency bias can be traced to contextual factors raters face (Tziner et al., 2005). For example, Uber riders reported giving higher ratings to drivers because they know drivers risk being de-registered if their average score drops below 4.6 (anything under the maximum five stars constitutes a failing grade) (Rosenblat and Stark, 2016). Heslin and VandeWalle (2008) found that a rater's implicit beliefs about people correlate with rater leniency. In addition to beliefs, at least one study found that cognitive ability is also related to leniency (Truxillo et al., 2008). Raters higher in cognitive ability tended to assign more severe ratings, while those lower in cognitive ability tended to assign more generous ratings. Banks and Murphy (1985) argued assigning ratings was unrelated to cognitive ability. Other sources of rating elevation could be dispositional and demographic characteristics. For example, people who score high in emotional-stability are more likely to provide unbiased ratings across dominant and friendly interpersonal settings, than people low in emotional-stability (Cheng, 2016). Agreeableness, extraversion, and emotional stability were related to performance ratings, and features of the rating context (e.g., study setting, appraisal purpose, accountability) moderated these relationships (Harari et al., 2015). Gender and race/ethnicity are related to magnitude of bias and to changes in bias across time (Tziner et al., 2005; Yun et al., 2005; Rosenman et al., 2011). More recently, a study of Airbnb Inc. found ratings are highly likely to be influenced by bias on the basis of factors like race or ethnicity. For example, guests with African-American names were about 16% less likely to be accepted as rentees than guests with characteristically white names (Edelman et al., 2016).

There has been some work on the effects of dispositional characteristics such as gender and self-monitoring. Tziner et al. (2008) reported a strong correlation between self-monitoring and leniency bias. This is understandable given the political considerations that often characterize such performance appraisal settings (Gioia and Longenecker, 1994; Latham et al., 2008; Hu and Liden, 2011). Jawahar (2001) found that the rater's attitude toward providing accurate performance feedback predicted accurate ratings for low self-monitors, but not for high self-monitors. Szarota et al. (2002) reported Dynamism and Excitability elicited higher agreement between self-and peer-ratings than Agreeableness and Intellect, although in case of Conscientiousness judges appeared to be as accurate as in the case of Excitability.

Wong and Kwong (2007) found that students who had been primed to maintain group harmony would provide an elevated evaluation of their peers. Bernardin et al. (2009) observed that those high on Agreeableness tended to rate the least effective performers more leniently than did other raters. People who were socially oriented, friendly, conscientious and concerned about other people's feelings tried not to make the target feel badly, even when a low rating was justified (Bernardin et al., 2009, 2016). Thus, raters who placed a high value on social interactions and sensitivity to colleagues' needs were less likely to be unduly harsh, since they would not want to risk their social ties at the workplace (Randall and Sharples, 2012). As Jawahar (2001) suggested, a concern for maintaining social relationships with others may contribute to leniency in rating others. This tendency would be strengthened if there is a concern not to hurt the self-esteem of the person being appraised. This would be found in someone who is socially sensitive and people-oriented. Being friendly, cheerful, and outgoing is a component of the extrovert personality. Based on this conceptualization and empirical findings, we expected more pronounced leniency bias from employees who have the motivation and energy to befriend others, and among those who are forgiving, understanding, approachable, and socially adept. To the extent that an agreeable and extrovert personality underlies these characteristics, we offer the following hypothesis:

H_1_: Leniency in rating others will be associated positively with the raters' Extroversion and Agreeableness.

### Possible causes of rating bias when rating oneself, and the role of personality

The psychological literature on leniency in self-rating uses a different set of labels, such as better-than-average effect, self-serving bias, self-deception, other-deception, self-enhancement, social desirability, and impression management (Wayne and Liden, 1995). Various explanations have been offered. For example, a cross-cultural perspective in individualism-collectivism (Farh et al., 1991; Xie et al., 2006; Heine and Hamamura, 2007; Cheng and Cascio, 2009; Ng et al., 2011) was found to be relevant in rating biases. Recently, there had been evidence supporting the contrary (Brown, 2010)—that is, while cultural factors may moderate leniency effect, the tendency to self-enhance is universal.

Robins and Paulhus (2001) postulated that personality traits as grandiosity and dominance served as underlying factors of self-enhancement. Farh and Dobbins (1989) found self-esteem higher among those who exhibited a tendency toward self-aggrandizement (which, unfortunately, in their study might have been confounded by true performance). Evidence exists that people who have considered themselves better than average have higher self-esteem and better mental health (see review by Taylor and Brown, 1994). Similar studies on self-enhancement and adjustment were reported by Kwan et al. (2008).

At the workplace, Murphy et al. (2004) reported raters purposely manipulated appraisal ratings in pursuit of their own goals. For example, they would over-report others' behaviors when seeking greater harmony with colleagues, or distort selected behaviors of executives when pursuing their own political goals. Sinha et al. (2012) also found self-raters high on extraversion, dominance, cultural conformity, cynicism, and detail orientation who were somewhat likely to over-rate their performance as compared to peer ratings.

Warr and Bourne (1999) reported that self-enhancement (i.e., self-ratings being higher than the ratings given by the supervisor) was associated with personality characteristics such as motivation to achieve. Conscientious and hardworking workers were more likely to observe and remember their own desirable work behaviors than those who did not pay much attention to their work. Goffin and Anderson (2007) reported similar findings. That is, inflated self-ratings of performance (relative to the ratings assigned by superiors) were associated with high achievement need. Both studies also found that the self-enhancement bias was weaker among people who were worrisome, anxious, and low in self-esteem (Warr and Bourne, 1999; Goffin and Anderson, 2007). Our second hypothesis, set in a Big-Five framework, attempts to replicate these findings:

H_2_: Leniency in rating oneself will be associated positively with Conscientiousness, and negatively with Neuroticism.

### Is there a general factor for rating bias?

In addition to evaluating the two hypotheses, we explored the relationship between self-rating leniency and other-rating leniency. Very few researchers have examined *in the same study* elevation bias in performance appraisal as well as self-enhancement and grandiosity. Besides the lack of a common metric for measuring the two types of leniency, another probable reason that such a study had previously not been carried out is that the research community does not see much conceptual linkage or psychological commonality between self-rating leniency and other-rating leniency. If there indeed were no substantively meaningful connections between the two rating types, we would observe a null correlation. This would imply that the elevation biases in self-ratings and other-ratings are driven by two different psychological processes.

This need not be true, however. Consider that both the tasks of rating oneself and rating others on a series of behavioral items require prior storage of behavioral episodes, retrieval of information from memory, and organization of the information for reporting. It is possible that some heuristics are used. If all leniencies are primarily a response set that people employ to do the rating task with minimal cognitive effort, self-rating leniency and other-rating leniency might utilize the same heuristics, and, consequently, would have some common associations. A positive correlation, therefore, should confirm this speculation and prompt a search for the common dispositional characteristic(s) that may underlie the general leniency bias. For exploratory purposes, we offer the following pair of competing hypotheses:

H_3a_: Other-rating leniency and self-rating leniency will not be associated with each other.
H_3b_: Other-rating leniency and self-rating leniency will be associated with each other, and also with certain common personality factors.

### Extension of past research

Our present investigation attempts to extend past leniency research in three ways: the operationalization of the construct; inclusion of peer raters; and a focus on a real-life field setting.

First, we attempt to operationalize leniency more accurately. One of the methods prior research has used is to ask participants to self-report their lenient tendency (e.g., Fried et al., 1999). Another approach we have taken is to treat as lenient those ratings that are distributed in a negatively skewed manner (Taylor and Hastman, 1956), that is, when raters assign a greater proportion of their ratings to the categories above their mean than to those below. However, without a sufficiently large number of ratings, it is not possible to determine skewness reliably. Even if there is skewness, at least part of it can be attributed to the skewed distribution of workers on their actual behaviors. Hence, unless the ratings were done on a scale anchored with statistically meaningful terms (e.g., a ratio scale based on magnitude estimation), and not with less precise ones (e.g., “very good,” “fairly satisfactory performance,” and so forth), it would not be justifiable to regard a high rating as lenient.

Another way to operationalize leniency is to compare the ratings against somebody else's judgment. This has been a method in many of the studies cited above. For example, in their study of students' bias in peer ratings, Bernardin et al. (2000) used the professor's rating as an estimate of a target student's performance. It should be noted, however, that such a measure inevitably confounds leniency with the target's actual performance, as well as with the professor's rating bias. In the present investigation, we derived a more objective measure of work performance without relying too much on the information provided by a single rater, who might have been equally biased. In doing so, the “leniency index” can be used to represent the psychological construct that we are interested in.

Second, we directed our attention to the self as raters. Most early work on rater accuracy used the immediate supervisor as the only rater. With the proliferation of 360° feedback, however, ratees as well as non-supervisors served as raters. This multi-rater procedure currently used in performance management and employee development rests on a rather strong assumption that ratings of behavior obtained from diverse sources are equally reliable and valid. Indeed, factorial structures of ratings scales have shown to be largely invariant across rater perspectives such as peer and self (Scullen et al., 2003; Hoffman and Woehr, 2009; Monahan et al., 2013). Nevertheless, researchers have also found discrepancies between self-ratings and ratings received from peers. For instance, self-appraisals have tended to be more lenient, less variable, more biased, and shown less agreement with the judgments made by others (Fox and Dinur, 1988; Diefendorff et al., 2005; Woehr et al., 2005; Hannum, 2007).

Despite the conceptual similarity between leniency in rating oneself and leniency in rating others, there is very little overlap between the two bodies of literature. For example, previous research has not compared whether one is more lenient with the self or with others. There is also no work to understand if the same set of psychological mechanisms operates upon the two leniencies. This is probably due to a lack of a common metric that researchers can use to index a person's leniency. To extend our knowledge, the present investigation examined how self-rating leniency and other-rating leniency might be associated with each other, and if leniency in these two sources could be ascribed to the same set of personality antecedents.

Third, the current study is a field study. A field study collects data outside a laboratory. Usually, it involves a range of well-defined variables in participants' natural environment (Cronbach, 1982). The strength of a field study is that it has ecological validity and demand characteristics are generally low. Field research is defined by its degree of naturalism (Reis and Gosling, 2010). Cronbach (1982) suggested when assessing the degree of naturalism of a study, one should refer to the units, treatments, setting and observations. As Paluck and Cialdini (2014, p. 82) illustrated, when studying the effects of interpersonal empathy, an effective way is to ask adults (not college undergraduates) to watch television episodes (not written instructions or simulated pictorials) with varying degree of emotionally arousing scenes while coding their facial reactions (instead of a written measure).

The present study meets the above criteria in four ways. First, the study sampled professional real estate agents in a developmental workshop. The second and third ways were that they were sampled based on service climate and personality, respectively. Fourth, during the workshop, the real estate agents completed a personality scale as well as a task to provide performance appraisal ratings to self and to their colleagues. Many have argued that ratings of performance pose threats to employees when done for promotional or remunerative purposes (Iqbal et al., 2015). The workshop hosts stressed that the ratings were for developmental purposes and that the data collected was not fed back to their employers.

One reason for the relative scarcity of published field studies in this area was that supervisors were dispersed in different work units, and consequently they rated their own staff, not others. Any observed difference between average ratings provided by two supervisors may suggest a difference in leniency, a genuine inter-unit difference in work performance, or both. While it is good research design to have two or more supervisors rating the same employee, often this is not feasible in field settings. Moreover, field settings often present difficulties for researchers who want to implement completely crossed experimental designs, in which each participant serves both as a rater as well as a ratee. In the present investigation, we extended previous laboratory studies by working with a sample that provided us with data that allowed us to compute leniency indices largely not confounded by participants' positions and roles. The sample is not large by conventional standards, but it allowed us to execute a meticulous procedure for data collection.

## Materials and methods

### Participants and procedures

In the field study, we collected data during a personal-development workshop for employees of an established real estate agency in Hong Kong. The attendees included 52 and 22 personnel in operational and managerial positions, respectively. For the operational staff, their average age was 33 years old. Those in managerial posts averaged 51 years old. The workshop discussed themes on personality at work, organizational climate, 360° rating systems and customer service quality. On completion of the workshop, the author and one master trainer consultant supervised an exercise designed to put participants' understanding of the above themes into practice. This included completing various scales related to the above themes. Role-plays on how quality service should be delivered also took place at this point.

Fifty-six male and eighteen female real estate agents rated themselves (i.e., self-rating) on a performance rating scale (to be described in criterion measures). Each agent was allowed up to five participants (including those in either operational or managerial posts) to provide ratings of their customer service performance (i.e., peer rating). Prior to the workshop the consultant specifically made arrangements with the agency's human resource personnel so that all participants were acquainted with at least three people. To ease possible concerns stemming from the evaluations, the hosts reiterated issues on anonymity and the University's policy on data privacy of participants. Participants were reassured the rating practice carried no administrative/remunerative consequences. Further, the ratings were to be aggregated and delivered anonymously and confidentially to the ratees from the hosts for purposes of personal development. This function was emphasized when the participants were instructed to choose as their raters those people who had had opportunities to observe them in action, and in whom they could trust for feedback. With this arrangement, all participants served both as raters and ratees. Two of the 74 participants were dropped from our sample because of incomplete data. The total number of observations in the study was 216. Each ratee had an average of 3.24 peer raters.

### Materials: personality measures

Personality was assessed on a Chinese translation of the 60-item NEO-FFI (Yik and Bond, 1993), with 12 items for each of the five dimensions. Cronbach's α (for the English version of the instrument) was 0.86 for the Neuroticism scale, 0.77 for Extroversion, 0.73 for Openness to Experience, 0.68 for Agreeableness, and 0.81 for Conscientiousness (Costa and McCrae, 1992). The Cronbach's α scores for the Chinese version on the same five scales were somewhat lower: 0.79, 0.65, 0.56, 0.49, and 0.75, respectively. As we did not develop a hypothesis on Openness to Experience, its reliability is not an issue here. Nonetheless, interpretation of any null effects for Agreeableness should be made with caution.

### Criterion measures

The real estate agent's job is primarily to facilitate transactions between a buyer and a seller, or between a landlord and a tenant. To devise an industry-appropriate measure of sales-oriented customer-service behavior, we asked a manager and five property dealers in the company to describe independently the daily routine of an agent, and what they considered to be the most important and desirable activities that a real estate agent should exhibit. Critical incidents were also solicited from the managers by three researchers, with education and training in industrial-organizational psychology. The interviews resulted in a summary of 16 behavioral statements agreed by the three researchers to reflect on contextual performance (for details of the item constructions, refer to Hui et al., 2003, 2007). As customer-service quality can be represented by how frequently these behaviors are displayed, we reformatted each item so that the frequency of the behaviors could be rated on a 1–5 Likert scale (1 = *never*, 3 = *occasionally*, 5 = *very often*). The Cronbach's α was 0.82 for self-rating, and 0.87 for rating of others. Sample statements were: “provides services not available from competitors,” “is able to negotiate a good price on customer's behalf,” and “makes customers feel that he/she is reliable and trustworthy.” For target persons (*n* = 15) rated by three peer raters, ICC was 0.03. For target persons rated by four and five peer raters, ICCs were 0.40 (*n* = 16) and 0.70 (*n* = 5), respectively. For target persons rated by six and seven peer raters, ICCs were 0.75 (*n* = 5) and 0.84 (*n* = 2), respectively. The low agreement among raters could be attributed to differences in contexts in which the target had been observed, but also to rater idiosyncrasies in leniency, which is the subject of this research. In the present study, we used as the true-score estimate the average of the peer ratings. We operationalized leniency as the elevation of the rating from the average of the ratings all others assigned to that same person. Since leniency for a given individual was defined relative to other people, the expected value of the overall mean of the leniency index was zero.

To recapitulate, in the present study three different sets of data were collected to investigate rater leniency. These included: measures of each participant's personality, his or her self-rating, and his or her ratings of several other people in the sample. From these data, we derived the following three measurement constructs:

1. One's actual performance, as averaged over all peer ratings assigned to that person. Understandably, ratings from different peer raters may not have coincided exactly with each other. The peers had seen the person in action at different times, and in different situations. In addition, each peer may have had his or her own bias. Furthermore, since the ratings came from people nominated by the target persons, errors of measurement would not be completely random. Nevertheless, the average peer rating appeared to be the best estimate of the target person's “actual” performance. It is at least better than the alternative, namely, ratings obtained from a set of randomly selected colleagues, whose variation in knowledge about the target persons may have introduced even more noise to the data and undercut external validity of any findings.
2. Other-rating leniency (ORL): this was the elevation of the ratings one assigned to a target person relative to the average ratings on the same target person by other peers, averaged across all targets that the rater has rated. This may be expressed algebraically as:
  (1) $$ORL_{ij}=\frac{\sum_{j\;=\;1}^{d}\sum_{i\;=\;1}^{d}R_{k}-\left(\frac{R_{1}\;+\;R_{2}\;+\;...\;+\;R_{p}}{p}\right)}{Nd}$$

where *ORL* = other-rating leniency; $\overset{d}{\underset{j=1}{\sum }}\overset{n}{\underset{i=1}{\sum }}R_{k}$ = ratings assigned to target persons *i* = *1* through *n* across dimensions *j* = *1* through *d* by rater *k*; *R*_1_, *R*_2_ … *R*_*p*_ = ratings assigned by raters *1, 2* through *p* of the same target person; *p* = the number of raters of each target person excluding rater *k*; *N* = the total number of target persons rated by rater *k*. Psychologically, this indicated how much more favorably this person rated others when compared to his or her fellow-raters.

3. Self-rating leniency (SRL): the elevation of ratings one assigned to oneself relative to the average ratings assigned by others to him or her. Algebraically:
  (2) $$SRL_{j}=\frac{\sum_{j\;=\;1}^{d}R_{k}-\left(\frac{R_{1}\;+\;R_{2}\;+\;...\;+\;R_{p}}{p}\right)}{d}$$

where all terms were as defined above.

## Results

Table 1 displays reliability estimates, means, and standard deviations of each of the personality scales, other major variables, as well as bivariate correlations among them. While the ratees' five personality scores did not correlate significantly with performance rating received from other raters, three (Neuroticism, Agreeableness, and Conscientiousness) correlated significantly with the ratees' self-rating. This strongly suggests common source problem and the possible impact of personality on the process of rating oneself.

**Table 1:** **Descriptive statistics of demographics, personality, and rating measures**.

	**Reliability**	**Mean**	**SD**	**Gender**	**Rank**	**N**	**E**	**O**	**A**	**C**	**RAO**	**RAS**	**RRO**	**ORL**	**SRL**
Gender (1 = male, 2 = female)		1.24	0.43												
Rank (1 = non-manager, 2 = manager)		1.28	0.45	−0.01											
Neuroticism (N)	0.79^a^	29.10	7.07	0.23	−0.15										
Extroversion (E)	0.65^a^	34.90	5.37	0.05	0.19	−0.42^***^									
Openness to experience (O)	0.56^a^	30.96	5.09	0.03	0.14	−0.09	0.10								
Agreeableness (A)	0.49^a^	28.92	4.96	0.05	−0.11	−0.26^*^	0.20	−0.05							
Conscientiousness (C)	0.75^a^	39.66	5.34	0.02	0.33^**^	−0.34^**^	0.22	0.06	0.34^**^						
Rating assigned to others (RAO)	0.40–0.70^b^	4.02	0.46	0.07	0.41^***^	−0.29^*^	0.40^***^	0.13	0.18	0.34^**^					
Rating assigned to self (RAS)	0.82^a^	4.07	0.42	0.09	0.35^**^	−0.30^**^	0.20	0.18	0.25^*^	0.33^**^	0.66^***^				
Rating received from others (RRO)	0.40–0.70^b^	4.05	0.35	0.10	0.62^***^	0.09	0.16	0.05	−0.11	0.04	0.30^**^	0.19			
Other-Rating Leniency (ORL)	–	−0.01	0.34	0.10	−0.03	−0.22	0.31^**^	0.13	0.24^*^	0.24^*^	0.82^***^	0.55^***^	−0.11		
Self-Rating Leniency (SRL)	–	0.02	0.49	0.01	−0.15	−0.32^**^	0.06	0.12	0.30^**^	0.26^*^	0.37^**^	0.72^***^	−0.55^***^	0.55^***^	
General Leniency Index	–	0.01	0.72	0.05	−0.11	−0.31^**^	0.19	0.14	0.31^**^	0.28*	0.63^***^	0.74^***^	−0.42^***^	0.83^***^	0.92^***^

N = 68–71; Standard error of correlation for ORL = 0.34; Standard error of correlation for SRL = 0.49;

a Cronbach's α;

b intra-class correlations for 4- to 5-person clusters;

* *p < 0.05 (2-tailed)*,

** *p < 0.01 (2-tailed)*,

*** *p < 0.001 (2-tailed)*.

In our sample, the difference between the rating assigned to oneself and the average rating assigned to others ranged from −0.85 to 1.17, with a median of 0.06. For 58% of the participants, the ratings they gave to themselves were higher than the average ratings they had given to others. Self-serving bias (i.e., rating oneself more favorably than rating others) existed in some, but not the majority of participants. Ratings assigned to self (*M* = 4.09, *SD* = 0.42, median = 4.06) were not different from ratings assigned to others (*M* = 4.02, *SD* = 0.46, median = 4.00). Nevertheless, these two ratings were highly correlated (*r* = 0.66, *p* < 0.001, *n* = 72, 95% C.I. = 0.52–0.79), suggesting individual differences in response sets such as leniency and extreme response style.

### Analyses on the leniency indices

SRL ranged from −1.06 to 1.03 (*n* = 71; *SD* = 0.49), and was more variable than ORL, which ranged from −0.74 to 0.89 (*n* = 72; *SD* = 0.34). The difference was statistically significant by Levene's test (*F* = 9.00, *df* = 1,141, *p* < 0.005). The two leniency indices were highly correlated (*r* = 0.55, *p* < 0.001, *n* = 69, 95% C.I. = 0.37–0.70), despite the fact that the average peer rating used for computing ORL was different from the one used to assess each participant's SRL. Partial correlations between the two indices with one of the Big Five traits controlled for each time ranged from 0.52 to 0.56 (*p* < 0.001, *df* = 65–66).

Our main hypothesis is that personality is related to leniency. Results indicated that ORL correlated with three of the Big Five personality traits: Extroversion (*r* = 0.31, *p* < 0.01, 95% C.I. = 0.08–0.51), Agreeableness (*r* = 0.24, *p* < 0.05, 95% C.I. = 0.01–0.45), and Conscientiousness (*r* = 0.24, *p* < 0.05, 95% C.I. = 0.01–0.45). SRL correlated negatively with Neuroticism (*r* = −0.32, *p* < 0.01, 95% C.I. = −0.52 to −0.09) and positively with Conscientiousness (*r* = 0.26, *p* < 0.05, 95% C.I. = 0.03–0.47) and Agreeableness (*r* = 0.30, *p* < 0.05, 95% C.I. = 0.07–0.50). We regressed ORL and SRL on the Big Five personality dimensions, after controlling for two demographic variables, gender and position (supervisory or otherwise). Results showed that the demographic variables did not have any effects on ORL, but neither did the personality factors. As for SRL, Neuroticism was a valid predictor (β = −0.30, *p* < 0.05).

Since there was a strong correlation between SRL and ORL, we aggregated ORL and SRL to form a general leniency index (GLI). We found that this new index correlated with Neuroticism (*r* = −0.31, *p* < 0.01, 95% C.I. = −0.51–0.08), Agreeableness (*r* = 0.31, *p* < 0.01, 95% C.I. = 0.08–0.51), and Conscientiousness (*r* = 0.28, *p* < 0.05, 95% C.I. = 0.05–0.49). In a hierarchical regression, after controlling for the effects of demographic variables, we identified two personality predictors: Neuroticism (*R*^2^ change = 0.13, *F* = 9.96, *df* = 1,66, *p* < 0.005, β = −0.29) and Conscientiousness (*R*^2^ change = 0.06, *F* = 4.51, *df* = 1,65, *p* < 0.05, β = 0.26). Emotionally stable and conscientious people were more generous in assigning their ratings. All of these findings were consistent with Hypothesis 3b, that there was a general leniency index. We also found in the same analysis that supervisors were more stringent than frontline staff (β = −0.25, *p* < 0.05)^1^.

### Regression analyses

In response to concerns raised about use of certain difference scores (Edwards, 1995), and to be conservative, we performed a parallel set of regression analyses without deriving any scores by subtracting one value from another. The criterion variables in these supplementary analyses were the ratings participants assigned, and not the leniency scores.

We conducted the first analysis, on ratings assigned to peers, using hierarchical linear modeling. This analytic technique allowed us to make simultaneous inferences on the effects of variations in the raters' personalities (at the individual, i.e., rater, level) and in the ratees' true performance (at the nested, i.e., ratee, level)^2^, which was statistically controlled. Table 2 contains the regression coefficients of the hierarchical regression analyses. At the ratee level, a substantial amount of variance in the ratings could be attributed to the true performance score. At the rater level, the model depicts the impact of two of the Big-5 personality dimensions. The raters' extroversion and conscientiousness contributed statistically significant variance to the criterion variable. This partially replicated results from analyses on ORL, thus lending additional support to Hypothesis 1.

**Table 2:** **Hierarchical regression analysis predicting peers' ratings assigned to target persons**.

		**Coefficients**	**Standard Error**	***p***
Level				
Rater	Gender	0.10	0.05	ns
	Seniority	−0.02	0.05	ns
	Neuroticism	0.01	0.00	ns
	Extroversion	0.01	0.00	0.001
	Openness to Experience	−0.00	0.01	cns
	Agreeableness	0.00	0.01	ns
	Conscientiousness	0.01	0.01	0.04
Ratee	Intercept	−0.70	0.30	0.02
	Actual performance	0.89	0.04	0.0005

We conducted the second series of hierarchical regression analyses to predict the ratings participants gave themselves. In the first step we entered the demographic variables as predictors, in the second step we entered the participants' true-scores, and in the third step we entered personality scores. Results showed that while demographic variables accounted for 12.9% of the variance in the criterion, adding the true score to the model did not improve the prediction of a person's self-rating. However, adding the participants' personality scores explained a significant amount of variance. The ΔR^2^ for Neuroticism was.08 [*F*_(1, 65)_ = 6.38, *p* < 0.05], for Agreeableness it was 0.08 [*F*_(1, 66)_ = 6.92, *p* < 0.05], and for Conscientiousness it was.05 [*F*_(1, 66)_ = 3.93, *p* = 0.052]. Adding an interaction term between personality and true score did not account for additional variability. This pattern is consistent with the findings for SRL, as reported in the previous subsection, and it confirms Hypothesis 2, that even after demographic variables and a person's actual performance have been controlled statistically, personality still has a substantial impact on one's self-rating.

## Discussion

This is the first study that used a common metric to assess employees' tendency to assign lenient ratings to self and to colleagues. Our basic premise was that raters' personalities might be related to leniency when they report on their own and others' work performance. We also considered the possibility that other-rating leniency and self-rating leniency might be affected by the same personality characteristic. Unlike most previous studies that investigated how the target person's self-rating differs from another person's (usually the supervisor's) rating, our study focused on the leniency operationalized by comparing the employee's judgment against an aggregation of ratings by several individuals, each of whom is reasonably knowledgeable about the target person.

Not everyone was generous in rating himself or herself, and at the same time harsh in rating others. This is consistent with the meta-analytic finding that the self-enhancement effect among Asians is not as strong as that among North Americans (Heine and Hamamura, 2007). There is, in fact, considerable inter-rater variability.

Results supported our general hypothesis that leniency can be traced, at least in part, to the rater's personality. It confirmed Hypothesis 1, that people who are outgoing, conscientious, and agreeable assign more generous ratings to others. People who are amiable tend to perceive a greater frequency of desirable behaviors in their peers' daily work. Furthermore, the study confirmed Hypothesis 2, that people who are emotionally stable and conscientious assign more generous ratings to themselves. Moreover, ORL and SRL are somewhat related to each other. However, controlling for personality did not attenuate the correlation between the two leniency indices. This suggests that any individual difference in *general* leniency in rating self and others could be due to characteristics beyond the Big Five.

### Personality correlates of ORL

Agreeableness and extroversion are the personality antecedents responsible for rating other people leniently. We can now speculate on the psychological mechanism that links these two traits to ORL. According to Murphy and Cleveland (1995), a rater may consider four possible goals: task performance (e.g., to raise or maintain target's task performance), interpersonal (e.g., to raise or maintain interpersonal relations with target), strategic (e.g., to increase target's chance of organizational advancement), and internalized (e.g., to rate according to some values and beliefs).

In the present investigation, where raters were not necessarily the targets' managers (they were mostly peers) and the purpose of rating was non-administrative, the first and third goals were not salient. As for the second, interpersonal goal, it is quite possible that the raters provided elevated ratings out of concerns about maintaining good relationships with the ratees. Given that our participants had been assured that their reports would not be seen by the target persons, there was minimal motivation for impression management. Other-rating leniency, reflecting a reluctance to deliver unfavorable observations to the recipients, could therefore be attributed to the person's genuine or habitual concern for others. Their internalized value to be friendly and caring manifested itself through their agreeable personalities, and resulted in favorable reports about their peers.

### Personality correlate of SRL

Our findings are consistent with previous research on the effect of self-esteem on self-rating. SRL was associated with low Neuroticism. People who are emotionally less stable (hence more likely to be low on self-esteem as well) were harsher when evaluating themselves than is warranted, while those who were emotionally stable tended to be more self-aggrandizing. It is possible that self-esteem and performance level were reciprocally related. People who were more confident, self-efficacious, and energetic had better work performance. Those who had a track record of high performance had built up positive self-esteem in various aspects of their lives, while people who had not achieved a high level of success may, as a result, feel less personal worth. Their low performance level was observable to raters, whose input we used to compute the average peer rating. The fact remains, however, that persons who had low self-esteem tended to assess their own performance even lower than their peers actually saw them. Conversely, the emotionally stable employees rated themselves higher than others rated them. Incidentally, it is worth noting that elevated ratings might still be psychologically useful.

The unspoken norm in most organizational settings is to manage others' (especially supervisors') impressions (e.g., Wayne and Liden, 1995). This involves, among other things, providing socially desirable responses in a self-appraisal. Lenient self-appraisals were used for impression management (to gain scarce rewards such as a promotion or an increase in salary), and to establish and maintain self-worth (see Paulhus, 1984). However, in the present research, where ratings were assigned confidentially and for a developmental purpose, and were not seen by company managers, SRL could be attributed only to the latter of these two motives. People generous in their self-reports thought highly of themselves (which is a sign of emotional stability), and people who thought highly of themselves tended to rate themselves more generously.

Could this correlation be spurious, with high SRL individuals who had inflated their own scores on desirable personality dimensions? It is not surprising that people with a positive view of themselves recalled more of their own positive work-related behaviors while completing a self-appraisal form, as well as recalled behaviors that were related to desirable traits while completing a personality inventory. The supplementary regression analysis also revealed the disturbing fact that one's self-rating on performance was not predicted by evaluations that others provided, but by one's own personality. Future research on this topic should therefore use alternative personality measures that do not rely upon self-reports, or at least instruments that are less susceptible to distortion motivated by needs for social and self-approval.

### Implications and limitations

With regard to the implementation of performance-management systems, Banks and Murphy (1985) questioned the efficacy of improving a rater's *ability* to judge performance. Our study echoes this by identifying an element in the rating process, namely the rater's personality characteristics, which may have nothing to do with ability. That one's self-ratings could not be predicted by the aggregated ratings received from peers, but could be predicted by one's own personality, adds to the ongoing controversy surrounding the accuracy and usefulness of this measurement (e.g., Atwater and Yammarino, 1997; Jones and Fletcher, 2002). A few limitations should be mentioned.

First, ratings in our study were assigned for developmental purposes only, while in organizational settings ratings have been usually assigned for administrative decision-making as well as for developmental purposes (Mount et al., 1998). Can findings from ratings assigned for developmental purposes be generalizable to those assigned for administrative purposes? Kraiger and Ford (1985) investigated a similar question in their meta-analysis and noted that the effects they found were not moderated by the administrative or developmental purpose of the ratings. In light of their findings, the generalizability concern might be relatively minor.

A second potential limitation is that while in real life supervisors provide most ratings, peers provided the ratings in the present study. A third potential limitation is that we assured participants of complete anonymity and confidentiality between the peer raters and the target persons. In performance appraisals other than 360° feedback programs, however, the boss (rater) usually has the responsibility to deliver the results to the target person face-to-face. Faced with such a situational imperative without the protection of anonymity, the boss may be under pressure to distort his or her ratings for a variety of political or motivational reasons (e.g., Longenecker et al., 1987; Gioia and Longenecker, 1994), none of which we have considered in this paper. These limitations may restrict the generalizability of our findings to peer, or 360° ratings, more so than to supervisory ratings. We therefore welcome more research in this direction.

A fourth limitation is that there is the usual concern whether findings from data collected in one part of the world is generalizable elsewhere. To the extent that the Big Five model and measurement of personality are applicable in both the Chinese sample and other world populations, and that the five scales correlated in a pattern similar to that found in previous American and European studies, the personality-leniency correlation may be generalizable beyond our present sample. Although, we consider it a strength that our data came from employees in a single organization (and therefore participants experienced a similar organizational culture), replications in other industries and countries are essential to establishing the robustness of this relationship.

A fifth and final potential limitation is the use of averaged peer ratings for computing the leniency indices. This procedure might possibly result in the loss of information about rater (dis)agreement (Sulsky and Balzer, 1988). However, because no objective measures of performance were available for many types of jobs, averaging is by far the best method to remove at least some measurement errors and systematic, rater-dependent biases. On the positive side, since the same contextual factors likely to promote or reduce leniency for a given rater also applied to other raters, any difference between the ratings assigned by that person and the ratings averaged from other people would primarily reflect dispositional factors and their interaction with situational factors, and not the situational factors alone.

## Ethics statement

The University of Hong Kong Research Committee. Participants took part in a development workshop commissioned by the stakeholder on personality and work performance. During the workshop, participants consented to the data being used for research purpose. They were informed that unless they consent to the use of their data, the data will be destroyed after suitable feedbacks are provided to them. All participants consented and signed the consent form.

## Author contributions

KC, CH, and WC are the sole authors of this research paper. Each author contributed equally to the work of this manuscript in all aspects.

## Funding

Innushuk consult is the fund provider for the publication fee.

### Conflict of interest statement

The authors declare that the research was conducted in the absence of any commercial or financial relationships that could be construed as a potential conflict of interest.

## Footnotes

^1^The above observation is partially supported by two discriminant analyses. In the first, we formed four groups by median-splitting the sample according to ORL and SRL. Extroversion discriminated the two harsh-on-others groups from the two lenient-on-others groups (Wilks' Lambda = 0.85, *F* = 3.74, df = 3.64, *p* < 0.05). The more sociable one is, the more likely that she or he will assign lenient ratings to herself or himself and to others. Thirty-five percent of the cases were classified correctly. In a second discriminant analysis, we formed three groups (those lenient on both self and others, those harsh on both self and others, and the rest). Neuroticism loaded on the discriminant function (Wilks' Lambda = 0.91, *F* = 3.41, df = 2.65, *p* < 0.05), and 48% of the cases were classified correctly.

^2^In HLM, the group-level variable (i.e., the true performance of the ratee) must, by definition, be a constant within each cluster of respondents reporting on the same ratee. Therefore, for this analysis we estimated “actual” performance by aggregating ratings received from all peer raters. Because of this, we expected a slight overestimation of the effect of this variable in the model.
